# The attention network test: a characteristic pattern of deficits in children with ADHD

**DOI:** 10.1186/1744-9081-4-9

**Published:** 2008-02-12

**Authors:** Steinunn Adólfsdóttir, Lin Sørensen, Astri J Lundervold

**Affiliations:** 1 Department of Biological and Medical Psychology, The Faculty of Psychology, University of Bergen, Jonas Lies vei 91, 5009 Bergen, Norway; 2 Centre for Child and Adolescent Mental Health, University of Bergen, John Lunds plass 3, 5020 Bergen, Norway

## Abstract

**Background:**

The Attention Network test (ANT) gives measures of different aspects of the complex process of attention. We ask if children with Attention Deficit Hyperactivity Disorder (ADHD) will show a characteristic pattern of deficits on this test.

**Methods:**

The sample included 157 children (M = 10 years) who performed the child version of ANT as participants of the Bergen Child Study. Children with an ADHD diagnosis (N = 45) were compared to a group of children with other diagnoses (N = 55) and a group of children without any diagnosis (N = 57).

**Results:**

The group of children with ADHD showed low accuracy scores and a variable response set, indicating an inattentive response style. No differences were found between the groups on RT and accuracy measures of the alerting, orienting, and conflict networks. A high correlation between full scale IQ (FSIQ) and ANT measures was only found in the ADHD group. When FSIQ score was included as a covariate, the group differences were not statistically significant on any ANT measure.

**Conclusion:**

The present study showed that accuracy and variability measures rather than measures of the three attention networks conveyed the characteristic pattern of deficits in children with ADHD. The results emphasized the importance of including these measures to extend the sensitivity of the ANT, and the importance of reporting results both with and without FSIQ as a covariate.

## Background

Attention is a complex cognitive function, dependent on interacting neural systems of the brain. According to the Attention Network theory the systems can be subdivided into an alerting or vigilance network, a network of orientation or selection, and an executive or conflict network [1]. A range of experimental, neuroimaging, and clinical studies have supported the theory [2-4] and Berger and Posner [5] as well as Fan et al. [6] have argued that the attention network model is of special interest in studies of attentional disorders, e.g. the Attention Deficit Hyperactivity Disorder (ADHD).

The three networks have been widely explored by using cue-target reaction time (RT) tasks [7] and tasks evoking a conflict (e.g. [8]). Recently, Fan, Posner and collaborators [6] developed an experimental task called the Attention Network Test (ANT), combining a cue-target and a flanker test to obtain measures of the efficiency and accuracy of the three networks. Recent studies have used different versions of ANT to study cognitive characteristics associated with ADHD. Booth [9] used the original child version and found no differences between children with ADHD and control children on any of the three networks. An Event Related Potential (ERP) study by Rodriguez [10] demonstrated a deviant ERP activation pattern on the alerting and conflict networks in young adults with the DSM-IV defined inattentive subtype of ADHD. A deviant activation pattern was also found in a Functional magnetic resonance imaging (fMRI) study by Konrad and colleagues [11]. This affected all three networks, but the behavioral data showed that only the conflict network was less efficient in ADHD children than in control children. These results suggest that the neural basis of the attentional networks may be affected in children with ADHD, even when this is not reflected in behavior measures.

Most studies using ANT have focused on the RT measures of the three attention networks, even though studies using measures from continuous performance tasks have shown that accuracy measures are more affected than RT measures in children with ADHD [12,13]. Furthermore, children with ADHD are shown to be impaired on measures of sustained attention and vigilance [12,14,15], they show a more variable RT and report more errors of omissions and commissions than their non-ADHD peers [16,17]. Most studies reporting such findings have used the Continuous Performance Test (CPT). A recent study by Oberlin et al. [18] included variability and error measures in their analysis of ANT results. They found that these measures discriminated adults with ADHD from controls. As far as we know, no study has generated error and variability measures from ANT in a study of children with ADHD.

The aim of the present study was to find characteristic patterns of ANT results in children with ADHD by including measures of error types and variability in addition to the conventional measures of the three attention networks (Table 1). From earlier studies we assumed that the conflict network was affected in children with ADHD. Furthermore, we expected the extended measures to add information about behavior characteristics of the ADHD group, i.e. we expected to find lower accuracy scores and higher response variability in children with ADHD than in their non-ADHD peers.

**Table 1:** Definition of variables

**Variable**	**Definition**
Reaction time	Mean reaction time (RT) for each cue and flanker condition
Hits	Number of correct responses
Overall errors	Number of overall errors
Wrong responses	All other error responses than omissions, perservations and outliers
Omissions	RT = 0 ms.
Perservations	RT > 0 ms. < 100 ms.
Outliers	Flanker × Cue RT - Stdev < 3*Stdev. RT = 0 can not be both omission and outlier and priority was given to omissions
Hits RT	Median RT for correct responses
Hits RT SE	Standard error of RT for correct responses. Measure of consistency of responses
Variability of SE	Standard deviation of the 3 standard error values calculated for each block. Measures within respondent variability
Hits RT block change	The slope of change in RT between blocks. Measure of vigilance
Hits SE block change	The slope of change in standard error of RT between blocks. Measure of consistency and vigilance
Attention Networks	Calculated both for RT and errors:
	Alerting = RT/error for no cue - RT/error for double cue
	Orienting = RT/error for central cue - RT/error for orienting cue
	Conflict: = RT/error for incongruent flanker - RT/error for congruent flanker

## Methods

### Participants

The present study is part of the Bergen Child Study (BCS). The protocol and population of the stages in the first wave of BCS are described in detail in separate publications [19,20], and only a short presentation will be given here. Briefly, the original BCS included three stages: screening for behavior problems and psychiatric disorder of the whole Bergen 7-9-year-old population using the Strengths and Difficulties Questionnaire (SDQ) [21], the Autism Spectrum Screening Questionnaire (ASSQ) [22], and items pertaining DSM-IV symptoms of ADHD and ODD from Swanson, Nolan, and Pelham, version IV (SNAP-IV) [23], supplemented with a number of items designed specifically for use in the BCS (*stage 1*); Development and Well-Being Assessment (DAWBA) [24] interviews with parents of children defined as screen positive in stage 1 and a sample of screen negative children (*stage 2*), and in-depth neuropsychiatric/neuropsychological assessment of a subsample of "DAWBA positive" (i.e. children who obtained a diagnosis according to DAWBA) and "DAWBA negative" children from *stage 2*. Children with a "chronic somatic disorder" reported by parents in *stage 1 *of the study [25] were included, regardless of participation in *stage 2*.

**Figure 1:**
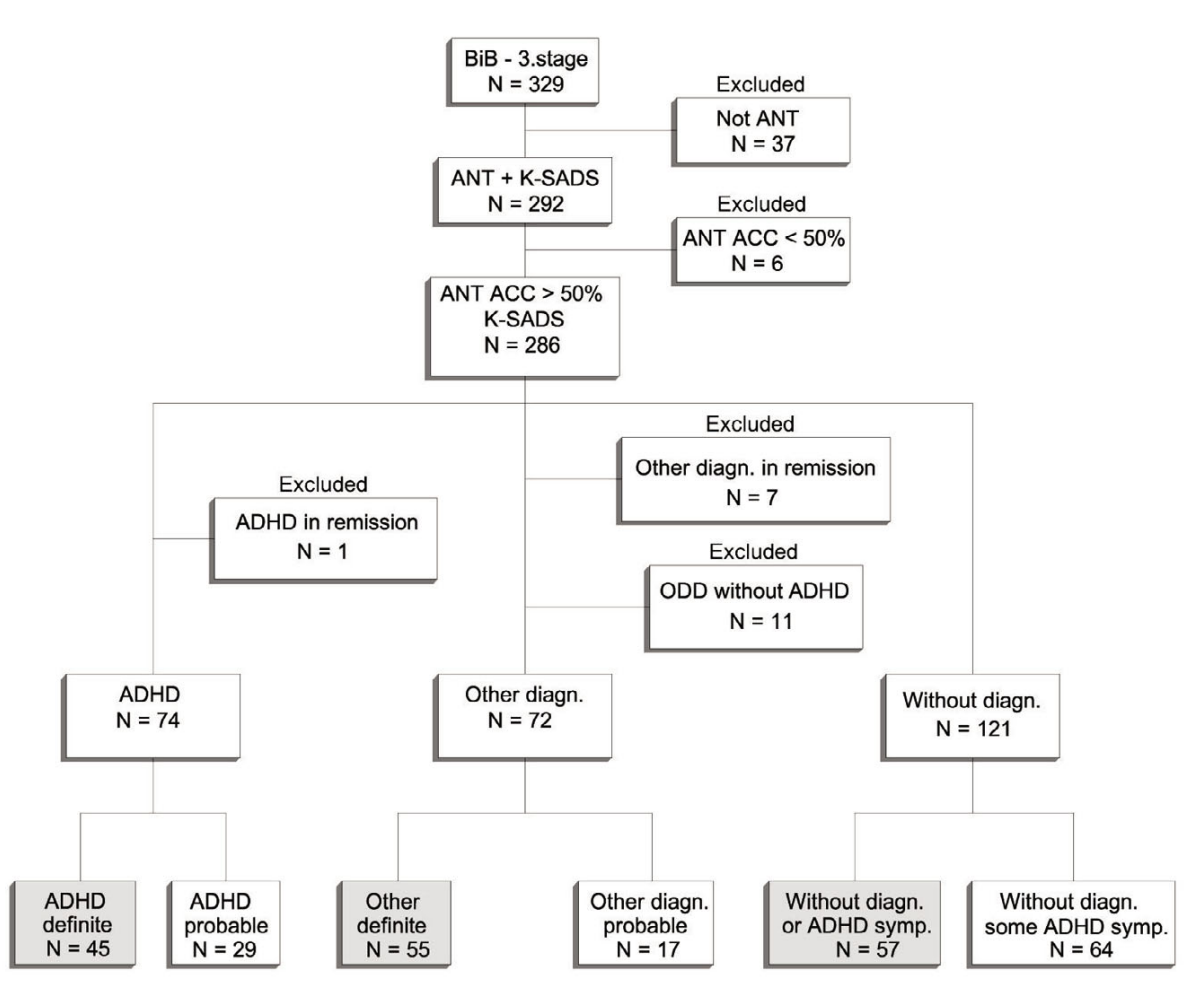
Flow chart visualizing the selection procedure.

The aim of *stage 3 *was to investigate the neuropsychological function (motor, emotional and cognitive) of children with known mental health problems and normal controls. A total of 329 children met together with their parents for a 6 hours examination procedure at the Neuropsychology outpatient clinic at the University of Bergen. The examination included a diagnostic semi-structured interview of parent and child (Kiddie-Sads-Present and Lifetime Version) [26]; the Wechsler's Intelligence Scale for Children, third version (WISC-III) [27], and the AANT [28]. Of the 286 children who completed the ANT with an accuracy scores above 50%, all children with a diagnosis in remission (N = 8) and the group of children with Oppositional Defiant Disorder (ODD) without ADHD (N = 11) were excluded. The last group was excluded due to the overlap in symptomatology. The final sample (N = 157) included all children with a definite ADHD diagnosis (N = 45), children with other definite diagnoses (N = 55), and children without any diagnosis or ADHD symptoms (N = 57) (Figure 1). The ADHD group was further divided into a group of children taking central stimulants at the inclusion of the study (N = 9) and children not taking any central stimulants (N = 36). The last group included mainly newly diagnosed children. All children taking central stimulants had an ADHD diagnosis at the entry of the study, and their parents were asked to withhold the medication on the day of clinical examination. Because of the low number of girls in the ADHD group (N = 13), gender differences were not investigated in the present study.

The study was approved by the Regional Committee of Ethics on Medical Research in Western Norway and by the Ombudsman for Privacy in Research, Norwegian Social Science Data Services Ltd.

### Measures

*Kiddie-Sads-Present and Lifetime Version (Kiddie-Sads-PL) *[26] was used as a diagnostic instrument. Kiddie-Sads-PL is a reliable semi-structured interview designed to evaluate current and past episodes of psychopathology in children according to the DSM-IV criteria [26,29]. The diagnosis was ascertained through an interview in two separate sessions on the same day, first with one or both of the parents and then with the child. Diagnoses were scored by the interviewer immediately after the assessment of both informants as either definite, probable (≥75% of symptom criteria met), in remission, or not present [26].

*The ANT *used in the present study is the original "child version" [30] downloaded from Jin Fan's webpage [28]. The test has four cue conditions (no cue, center, double, orienting) and three flanker conditions (congruent, incongruent, neutral), and has been described in detail elsewhere [30,31]. All combinations of conditions are randomly presented in three blocks of 48 trials each. Overview of the calculations used in the present study is given in Table 1. The calculated measures are based on an Excel macro downloaded from Jin Fans webpage [28], supplemented by measures calculated according to the formulas given by Conners and collaborators [32]. This file was imported into SPSS 13.0, which was used for all further analyses.

*WISC-III *[27] was used to assess intellectual function. The Full Scale IQ (FSIQ) was included in the present study, with scaled scores derived from Swedish norms [33].

### Procedure

The clinical examination was performed in an outpatient clinic at the University of Bergen. Trained psychologists administered the Kiddie-Sads-PL interview. The WISC-III and the ANT were administered by trained test-assistants, in a quiet room designed for testing. It was run on E-Prime software, on a stationary computer with a 17" computer screen. The children sat at a comfortable distance from the screen and used left and right thumb to press the left or the right mouse button, corresponding to a left and right pointing fish. The children were instructed to help feed the hungry fish as fast as they could by pushing the left or right button, according to which direction the fish was pointing. They were told that sometimes the fish would appear alone, and other times it would swim together with other fishes. In all cases, they were told to concentrate on the fish in the middle. They were also asked to keep their eyes on the fixation point during the presentations. The completion time was approximately 25 minutes.

### Statistical analysis

A Pearson correlation analysis with Bonferroni correction was computed, including the FSIQ score, age, and ANT measures. For FSIQ score, age, network measures, error measures, RT, standard error (SE) of RT, variability of SE, and vigilance measures, separate one-way between-groups ANOVAs were calculated. Main effects were further explored with post-hoc tests. The Tukey HSD was used to explore differences between groups where equivalence of variances were assumed, and the Games-Howell test was used if variances were heterogeneous. Bonferroni corrected independent-samples t-test was used to compare the results of children in the ADHD group who regularly took central stimulants and the results of the non-medicated children in the same diagnostic group.

## Results

### Age and FSIQ scores

The age range of the participants was 7.9 to 11.9 years, with a mean age of 10. A one-way ANOVA showed a statistically significant difference between the three diagnostic groups in age, *F*(2, 154) = 3.311, *p *= .039, but Tukey HSD between group comparison revealed that the difference between the ADHD group and the other groups only bordered on significance (other diagnoses: *p *= .07; without any diagnosis: *p *= .06). The mean FSIQ score for all participants was 90.9 (Table 2). A one-way ANOVA revealed a statistically significant difference between the groups on the FSIQ measure, and a Games-Howell post-hoc test showed that the ADHD group obtained significantly lower FSIQ scores than the non-ADHD groups (Table 2 and 3).

**Table 2:** Means and SDs of the ADHD and non-ADHD groups on demographic variables and selected ANT variables.

	ADHD definite diagnosis N = 45	Other definite Diagnoses N = 55	Without diagnosis N = 57	Overall N = 157
	
	RT/Error M (SD)	RT/Error M (SD)	RT/Error M (SD)	RT/Error M (SD)
**FSIQ and age**				
FSIQ	78,7 (17,6)	93,6 (15,2)	97,8 (11,5)	90,9 (16,7)
Age	10,3 (0,8)	9,9 (1,0)	9,9 (0,9)	10,0 (0,9)
**Accuracy and error variables**				
Hits	125,1 (16,7)	132,4 (8,6)	133,1 (9,7)	130,6 (12,2)
Overall errors	18,9 (16,7)	11,6 (8,6)	10,9 (9,7)	13,4 (12,2)
*Wrong response*	5,4 (4,5)	3,3 (3,3)	3,2 (3,3)	3,9 (3,8)
*Omissions*	12,9 (14,6)	7,9 (7,1)	7,3 (7,9)	9,1 (10,3)
*Perservations*	0,2 (0,5)	0,1 (0,3)	0,1 (0,2)	0,1 (0,4)
*Outliers*	0,3 (0,6)	0,3 (0,5)	0,3 (0,8)	0,3 (0,7)
**Consistency and variability variables**				
Hits RT	828,3 (148,4)	791,9 (123,7)	791,8 (124,7)	802,3 (131,8)
Hit RT SE	22,5 (5,6)	20,2 (4,1)	19,7 (5,0)	20,7 (5,0)
Variability of SE	5,2 (2,9)	5,4 (2,4)	5,3 (3,8)	5,3 (3,1)
Hit RT Block Change	-3,1 (48,9)	-27,8 (52,7)	-13,9 (66,7)	-15,7 (57,7)
Hit SE Block Change	0,3 (4,9)	-0,1 (4,9)	0,5 (5,4)	0,2 (5,1)
**Networks – RT**				
Alerting	109,2 (81,6)	101,0 (63,6)	80,8 (74,4)	96,0 (73,5)
Orienting	27,7 (60,6)	37,5 (59,3)	32,0 (58,7)	32,7 (59,2)
Conflict	92,2 (85,6)	81,0 (60,2)	87,3 (56,1)	86,5 (66,9)
**Networks – Errors %**				
Alerting	2,6 (9,9)	2,4 (5,1)	3,3 (6,6)	2,8 (7,3)
Orienting	0,3 (7,0)	0,3 (5,9)	0,2 (5,7)	0,3 (6,1)
Conflict	4,6 (11,2)	4,4 (7,4)	3,4 (6,1)	4,1 (8,2)

**Table 3:** A one-way ANOVA showing the difference between the groups on selected ANT variables.

	Main effect of between groups ANOVA		
	
	*Df*	*F*	*Between*
**Covariate**			
FSIQ	2, 154	22.66***	1 < 2,3
**Accuracy and error variables**			
Overall errors	2, 154	6.77**	1 > 2,3
*Wrong respons*	2, 154	5.58**	1 > 2,3
*Omissions*	2, 154	4.54*	*ns*
**Consistency and variability variables**			
Hits RT	2, 154	1.23	*ns*
Hit RT SE	2, 154	4.41**	1 > 3
Variability of SE	2, 154	.05	*ns*
Hit RT Block Change	2, 154	2.34	*ns*
Hit SE Block Change	2, 154	.22	*ns*
**Networks**			
Alerting – RT	2, 154	2.09	*ns*
Orienting – RT	2, 154	.34	*ns*
Conflict – RT	2, 154	.35	*ns*
Alerting – Error	2, 154	.21	*ns*
Orienting – Error	2, 154	.01	*ns*
Conflict – Error	2, 154	.34	*ns*

* = p < .05 ** = p < .01 *** = p < .001

Group: 1 = ADHD; 2 = Other diagnoses; 3 = No diagnosis

### Attention Networks

A one-way between groups ANOVA revealed no statistically significant differences between the three diagnostic groups on the RT and error measures of the three attention networks (Table 2 and 3).

### Error measures

The overall accuracy on the ANT was 90.7%, and an analysis of the overall number of errors showed that 97.0% of all errors were wrong responses and omissions (Table 2). Outliers and perservations represented only 3.0% of all errors and less than 1% of all responses, and were not included in further analyses. A one-way between groups ANOVA of the remaining error measures showed a statistically significant main effect of group (Table 3). A Games-Howell post-hoc test revealed statistically significant higher overall error scores in the ADHD group than the non-ADHD groups, with significantly more wrong responses than the non-ADHD groups (Table 2 and 3). The number of omission errors was also higher in the ADHD group than in the non-ADHD groups, but the differences were not statistically significant (compared to the group with other diagnoses, *p *= .09 and the group without any diagnosis, *p *= .06).

### Variability measures

A one-way between groups ANOVA was performed separately for the overall Hit RT and the variability measures (Hit RT SE, variability of SE, Hit RT block change, Hit SE block change). A statistically significant main effect of group was found on the Hit RT SE measure (Table 2 and 3). Between group Tukey HSD comparisons revealed that the ADHD group showed significantly larger Hits RT SE than the group without diagnosis and border on significance when compared to the group with other diagnoses (*p *= .054). No main effect of group was found on the other measures.

### Age and FSIQ scores correlation

A Bonferroni corrected Pearson's correlation analysis was performed between FSIQ scores, age and ANT measures (Table 4). The analysis revealed a high correlation between age and the overall Hit RT and the variability measures, showing that the older the children, the faster they responded, with a higher level of consistency. Table 4 shows that this only applied to the two non-ADHD groups. Correlations between FSIQ scores and the ANT measures were more widespread, including both error and variability measures. Table 4 shows that this only applied to the ADHD group, showing that the lower the FSIQ scores, the less accurate the children responded and with less consistency.

**Table 4:** Correlations between selected ANT variables within the three groups and for all participants.

	ADHD definite diagnosis N = 45		Other definite Diagnoses N = 55		Without diagnosis N = 57		Overall N = 157	
	
	Age	FSIQ score	Age	FSIQ score	Age	FSIQ score	Age	FSIQ score
**Accuracy and error variables**								
Overall errors	-.167	-.509**	-.298	-.255	-.314	-.294	-.164	-.455**
*Wrong response*	-.031	-.497**	-.111	-.149	-.264	-.152	-.073	-.373**
*Omissions*	-.191	-.408*	-.315	-.236	-.274	-.277	-.174	-.388**
**Consistency and variability variables**								
Hits RT	-.164	-.309	-.469**	-.101	-.411**	-.183	-.320**	-.235*
Hit RT SE	-.094	-.472**	-.321	-.239	-.235	-.207	-.159	-.381**
Variability of SE	.051	-.084	.181	-.054	.066	-.036	.089	-.041
Hit RT Block Change	.083	.069	.287	-.176	.415**	.098	.312**	-.058
Hit SE Block Change	.034	-.108	.119	-.240	.299	.009	.164	-.100

* = Correlation is significant at the 0.05 level 2 tailed

** = Correlation is significant at the 0.01 level 2 tailed

### FSIQ score as covariate

When FSIQ was included as a covariate in the ANOVA analyses, the main effect for group was no longer statistically significant for the overall error measure (*F*(2, 153) = 0,689, *p *= .504) and the Hit RT SE measure (*F*(2, 153) = 0,316, *p *= .729).

### Influence of medication

The group of ADHD children taking central stimulants regularly (N = 9) was compared to the other ADHD children (N = 36) on selected ANT measures (overall errors, Hits RT, Hits RT SE) and FSIQ scores. Bonferroni corrected independent-samples t-test showed no statistically significant difference between the two groups.

## Discussion

The aim of the present study was to characterize patterns of ANT performance in children with an ADHD diagnosis. On the measures of the three attention networks, the results revealed no statistically significant differences between the group of children with ADHD, the group of children with other psychiatric diagnoses and the group of children without any diagnosis. However, the children diagnosed with ADHD showed a lower accuracy score as well as a more variable response pattern (i.e. a higher SE of RT) than the other groups.

The results confirmed the expectation of lower accuracy scores in the ADHD group compared to what was shown in the other two groups. The ADHD group reported more wrong responses and showed a trend towards more omissions errors compared to the non-ADHD groups. The high number of omission errors in the ADHD group indicated a higher level of inattention than in children belonging to the two other groups, a finding that confirms earlier findings in studies using the Conners' CPT [16,17]. The results did also support the prediction of high response variability in the ADHD group. However, the variability of SE was not different between the three groups. According to Conners [32], a pattern of higher Hits SE RT than Variability SE suggests a poor consistency of responses that did not change as the test progressed. This supports the idea that problems related to inattention rather than vigilance are characteristic of children with ADHD.

Our findings with respect to vigilance are in conflict with results from studies using CPT, suggesting that loss of vigilance as the test progresses is characteristic of children with an ADHD diagnosis [12,14,15]. It is well known that children perform better on tasks with a vivid feedback and on tasks that have an underlying story [30]. The child version of the ANT is more similar to a computer-game with immediate and clear feedback on performance than the Conners' CPT. The ANT includes a character (the fish), a narrative (is hungry, help feed him), and auditory and visual feedback (fish blowing bubbles and wagging its tail as well as exciting sound), and these features have been found to improve the performance on more game-like versions of the CPT [34]. This and the fact that the ANT has just three time blocks compared to six in the CPT, may have made the vigilance measure less sensitive to a core problem of children with ADHD [12,14-16].

The FSIQ score was strongly correlated with both error and variability measures, but only in the ADHD group. When included as a covariate, all differences between the ADHD group and the two other groups became non-significant. There is an ongoing debate whether or not one should control for IQ in studies of cognitive function in children with ADHD [15,35-37]. Several studies have shown that children with ADHD tend to obtain lower IQ scores than other children [35,37], and that the neurocognitive disorders of ADHD in itself can cause poor performance on intelligence tests [38]. Actually, a meta-analysis found a strong association between ADHD and FSIQ (*d *= .61) [35]. This is supported by the results in the present study, showing that the highest and most widespread correlations were found in the ADHD group. If reduced IQ is a developmental consequence of the ADHD disorder, then, by controlling for IQ, one may very well control for a part of the disorder [35,36]. This has led Barkley [38,39] to argue that it is probably unwise to control for IQ score in studies comparing ADHD groups and controls, and that studies of ADHD should rather report results with and without controlling for IQ scores [40], as done in the present study.

While the IQ scores showed the strongest associations with errors, age was more strongly associated with the Hit RT and variability measures. Age was correlated with faster overall RT in the non-ADHD groups, confirming earlier findings that RT improves with age [30,31]. However, age was not significantly correlated to any of the dependent ANT measures in the ADHD group, suggesting that children with ADHD do not show the expected improvement of RTs as they age. The results revealed no significant group differences on the efficiency and error measures of the three attention networks. These results are in accordance with Booth's [9] findings in a study using the same child version of the ANT as in the present study. However, the results did not support the findings of Konrad et al. [11], who showed a significant deficit in the efficiency of the conflict network. One explanation may be that they used a modified ANT procedure. Rueda et al. [30] found that the fish target used in the present study as well as a paradigm including only valid cues generates a smaller interference effect of incongruent flankers than the arrow target. This implies that the paradigm used in the present study may have made it easier for the children to solve the conflict between the congruent and incongruent flankers than in the study of Konrad and collaborators [11], and may indicate a need for revision of the child version of the ANT in future studies of children with an ADHD diagnosis.

Based on the behavioral measures of the attention networks, one should not exclude the possibility of a characteristic neural activity in children with ADHD, as suggested by Rodriguez [10] and Konrad et al. [11]. From these studies one may argue that children with ADHD use different strategies for completing tasks than their peers, and that behavioral measures are not sensitive enough to detect this difference [11]. However, the high number of errors reported by children with ADHD in the present study may be used to support the idea of a less effective use of strategies in children with ADHD than in their non-ADHD peers.

### Strengths and weaknesses

As in all research including children with an ADHD diagnosis, the present results are colored by the high degree of heterogeneity within the diagnostic group. In the present study, information about subgroups of ADHD and symptom load was not included. Both Booth [9] and Rodriguez [10] found a difference between the DSM-IV defined diagnostic subgroups of ADHD on the network measures. On the other hand, Seidman [36] argues that there are more similarities between the subgroups of ADHD than dissimilarities when it comes to measures of cognitive functions. We have calculated the within response variability according to Conners [32]. According to Russell et al. [41], more extended calculations may give more adequate measures of variability and should be considered in further studies.

The main strength of the study was the case-control selected sample of children with ADHD, and that the results probably are less biased by co-morbid problems than clinical studies. However, no child was excluded due to a low FSIQ score, although some of the BCS participants with very low FSIQ score were excluded because they were unable to perform the ANT. The high correlations between the FSIQ score and error and variability measures in the ADHD group indicate that by excluding children with low total IQ scores, one may have excluded a specific group of ADHD children [39].

### Clinical implications

Although there have been several studies of the neuropsychological characteristics and the neural basis of ADHD, the deficits of attention in children with this behavioral diagnosis are still poorly understood. To conduct studies of this complex issue, appropriate neurocognitive models that operationalize different aspects of the attention system are necessary. The attention network theory provides one such model and can be used both in group studies and in the clinical evaluation of individual children. In a neuropsychological examination, the range of variables from the ANT may help to characterize the strengths and difficulties of a child. Studies of the attention networks in children with ADHD may contribute to a better understanding of the disorder and to the development of appropriatetraining and treatment methods [42].

## Conclusion

The results in the present study support the notion that accuracy measures rather than RT measures are sensitive to characteristic deficits in children with ADHD [12,13]. The results also demonstrate the importance of including accuracy measures and variability measures to extend the sensitivity of the ANT to deficits that characterize children with an ADHD diagnosis. Nevertheless, there is a need of developing the test measures, and to perform studies investigating the clinical significance of the errors and variability shown by children with ADHD.

## Competing interests

The author(s) declare that they have no competing interests.

## Authors' contributions

SA has been responsible for the data analysis and the writing of the manuscript. AJL designed and coordinated the study, supervised the data analysis and the writing process. LS participated in the acquisition of data, discussions about the data analyses and commented on the written drafts of the manuscript. All authors have read and approved the final manuscript.

## List of abbreviations

ADHD: Attention Deficit Hyperactivity Disorder; ANT: Attention Network Test; BCS: Bergen Child Study; CPT: Continuous Performance Test; FSIQ: Full Scale IQ; ODD: Oppositional Defiant Disorder; RT: Reaction time; Kiddie-Sads-PL: Kiddie-Sads-Present and Lifetime Version; WISC-III: Wechsler Intelligence Scale for Children-III.
